# Effects of cholinesterase inhibition on attention and working memory in Lewy body dementias

**DOI:** 10.1093/braincomms/fcad207

**Published:** 2023-07-27

**Authors:** Sean James Fallon, Olivia Plant, Younes A Tabi, Sanjay G Manohar, Masud Husain

**Affiliations:** Department of Experimental Psychology, University of Oxford, Oxford OX2 6GG, UK; School of Psychology, University of Plymouth, Plymouth PL4 8AA, UK; Department of Experimental Psychology, University of Oxford, Oxford OX2 6GG, UK; Department of Experimental Psychology, University of Oxford, Oxford OX2 6GG, UK; Department of Experimental Psychology, University of Oxford, Oxford OX2 6GG, UK; Nuffield Department of Clinical Neurosciences, John Radcliffe Hospital, Oxford OX3 9DU, UK; Department of Experimental Psychology, University of Oxford, Oxford OX2 6GG, UK; Nuffield Department of Clinical Neurosciences, John Radcliffe Hospital, Oxford OX3 9DU, UK

**Keywords:** Lewy body dementias, sustained attention, working memory, acetylcholine, cholinesterase inhibitors

## Abstract

Cholinesterase inhibitors are frequently used to treat cognitive symptoms in Lewy body dementias (Parkinson’s disease dementia and dementia with Lewy bodies). However, the selectivity of their effects remains unclear. In a novel rivastigmine withdrawal design, Parkinson’s disease dementia and dementia with Lewy bodies patients were tested twice: once when taking rivastigmine as usual and once when they had missed one dose. In each session, they performed a suite of tasks (sustained attention, simple short-term recall, distractor resistance and manipulating the focus of attention) that allowed us to investigate the cognitive mechanisms through which rivastigmine affects attentional control. Consistent with previous literature, rivastigmine withdrawal significantly impaired attentional efficacy (quicker response latencies without a change in accuracy). However, it had no effects on cognitive control as assessed by the ability to withhold a response (inhibitory control). Worse short-term memory performance was also observed when patients were OFF rivastigmine, but these effects were delay and load independent, likely due to impaired visual attention. In contrast to previous studies that have examined the effects of dopamine withdrawal, cognitively complex tasks requiring control over the contents of working memory (ignoring, updating or shifting the focus of attention) were not significantly impaired by rivastigmine withdrawal. Cumulatively, these data support that the conclusion that cholinesterase inhibition has relatively specific and circumscribed—rather than global—effects on attention that may also affect performance on simple short-term memory tasks, but not when cognitive control over working memory is required. The results also indicate that the withdrawal of a single dose of rivastigmine is sufficient to reveal these impairments, demonstrating that cholinergic withdrawal can be an informative clinical as well as an investigative tool.

## Teaser

Fallon *et al.* tested sustained attention and short-term memory in Lewy body dementia patients on two occasions: once whilst taking rivastigmine as usual and once when they had missed one dose. Patients had superior sustained attention and short-term recall ON compared to OFF rivastigmine. Distractor resistance was unaffected.

## Graphical Abstract

**Graphical abstract:**
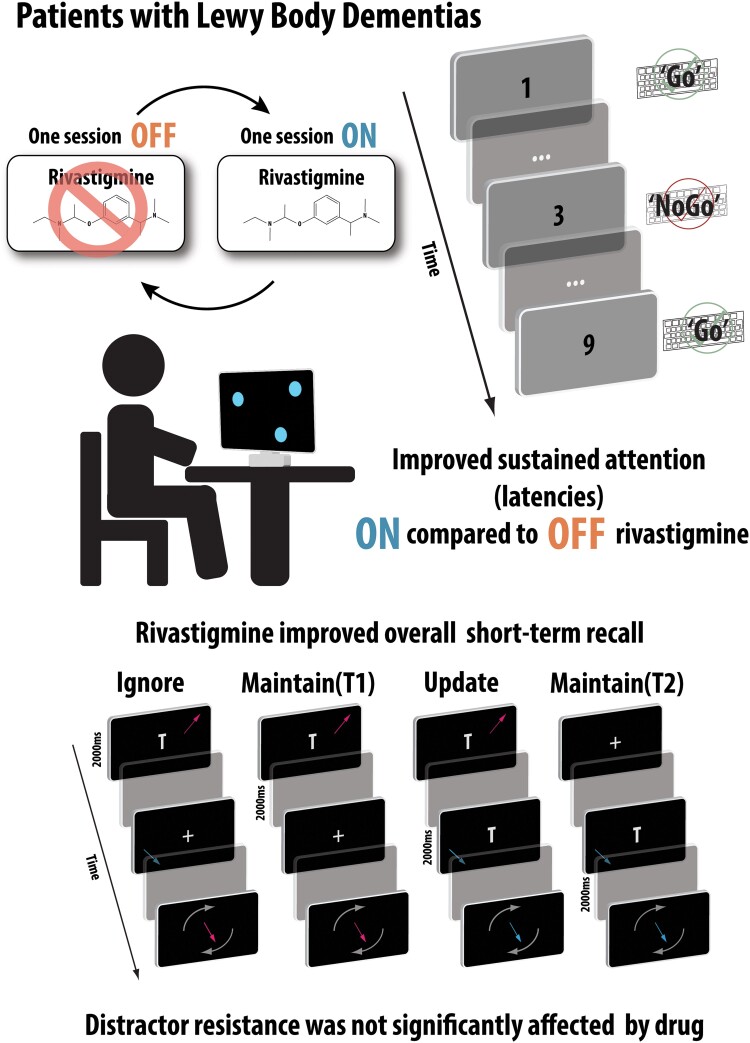

## Introduction

Parkinson’s disease dementia (PDD) and dementia with Lewy Bodies (DLB) are now understood to be very closely related conditions, both associated with Lewy body pathology and manifesting with cognitive and motor symptoms along a continuum.^1^ Although the constellation of pathological changes triggered by these two neurodegenerative conditions is complex and encompasses disruptions to many neurotransmitter systems,^2–4^ cholinergic decline across the cortex is particularly prominent.^5^ Clinical trials have shown that PDD and DLB patients prescribed with cholinesterase inhibitors, such as rivastigmine, show modest improvements in global cognitive performance.^6–8^ However, the cognitive domains that cholinergic augmentation can reprieve has not been fully mapped.

Many lines of existing evidence strongly support the notion that cholinesterase inhibitors—and remediations to acetylcholine levels in general—improve attention across a variety of clinical conditions.^9–11^ For example, administration of rivastigmine reduced the reaction time variability after 24 weeks in both PDD and DLB patients.^11^ This is consistent with mechanistic work in healthy human participants that highlight the importance of the cholinergic system in being able to register and respond consistently to environmental cues.^12,13^ Indeed, at present, there is limited evidence that cholinesterase inhibition in clinical groups improves cognitive functions outside, or independently, of attention.^14^

This raises questions regarding attempts to ameliorate other cognitive symptoms, e.g. deficits in short-term memory that form a core part of Lewy body dementias and contribute to distress and reduced quality of life.^15,16^ Given the extensive cholinergic innervation of cognitive control circuits in the brain and the negative effects of cholinergic perturbations on short-term memory,^13,17–19^ mnemonic gains after rivastigmine might nevertheless be expected. One possibility is that the drug affects certain sub-components of short-term or working memory, and not others.

Indeed, the heterogenous nature of the neurocognitive mechanisms involved in the short-term retention of information has long been recognized.^20–23^ Specifically, researchers have posited a multiplicity of cognitive mechanisms and neural substrates involved in maintaining information across longer delay periods,^24,25^ set size,^26,27^ requirement to ignore irrelevant information,^28,29^ or shift attention between items in memory.^30,31^ Dopamine has been argued to have highly nuanced and task-specific effects on working memory, with a prominent role for this neurotransmitter in supporting the control over the contents of mnemonic representations.^32,33^ Withdrawing PD patients from their dopaminergic medication does not affect the ability to maintain information but does impair cognitive control over retained items, for example, re-arranging the serial order of information^34^ or ignoring and updating items in working memory.^28^ Thus, it is theoretically possible that cholinesterase inhibition might have analogously selective effects on working memory sub-processes via modulating distinct cholinergic pathways in the brain.

Here, we seek to fractionate the effects of cholinesterase inhibition on sustained attention and various putatively distinct variables in short-term and working memory (delay, load and manipulating irrelevant information) in people with Lewy body dementias. We used a novel withdrawal design in which we attempt to isolate the effects of rivastigmine on cognition in an analogous fashion to that previously performed in dopamine withdrawal studies.^35,36^ We tested patients twice: once when taking their rivastigmine as usual (ON condition) and once when they had skipped their most recent dose (OFF condition). This is possible because orally administered rivastigmine has a short plasma elimination half-life (1–2 hours), and inhibiting acetylcholinesterase for ∼10 hours,^37^ permitting the cognitive effects of withdrawal to be observed.

## Methods

### Participants

Patients were recruited from the Oxford University Hospitals Cognitive Disorders clinic between November 2017 and August 2018. Consecutive patients who attended clinic with a diagnosis of idiopathic PDD or DLB (diagnosed when the onset of cognitive impairment occurred prior to 1 year after the onset of motor signs) were screened. Twenty-two patients who were taking rivastigmine were recruited. This number would give us power (*n* = 16, effect size = 0.77, 80% power, alpha = 0.05, non-parametric pairwise comparison) to detect similar effects to previous studies,^38^ allowing for possible attrition.

Mean age of participants was 64.3 ± SD 6.8 years. Addenbrooke’s Cognitive Examination (ACE) III^39^ tests were administered in the ON and OFF sessions. One patient was missing an OFF score. For the remaining patients (19 male; 2 female), the mean total ACE score ON rivastigmine was 84.1 ± 6.2 and 81.8 ± 6.2 OFF rivastigmine (*t*(19) = 1.75, *P* = 0.09). Participants had a mean of 14.8 ± 4.7 years of education and all but one were right-handed, with normal or corrected-to-normal vision. The mean total Unified Parkinson's Disease Rating Scale (UPDRS) score was 64.8 ± SD 33.7 (performed during the ON session). Five patients were not taking any dopaminergic medication. The average levodopa equivalent dose^40^ was 585 ± 427 mg, which was mainly in the form of levodopa, with three patients on ropinirole and two patients on pramipexole. Seven patients were also on selective serotonin reuptake inhibitors (SSRIs), and seven were on benzodiazepines for sleep disorder. Five individuals were on pregabalin for anxiety.

#### Tasks

##### Sustained attention to response task

**Figure 1:**
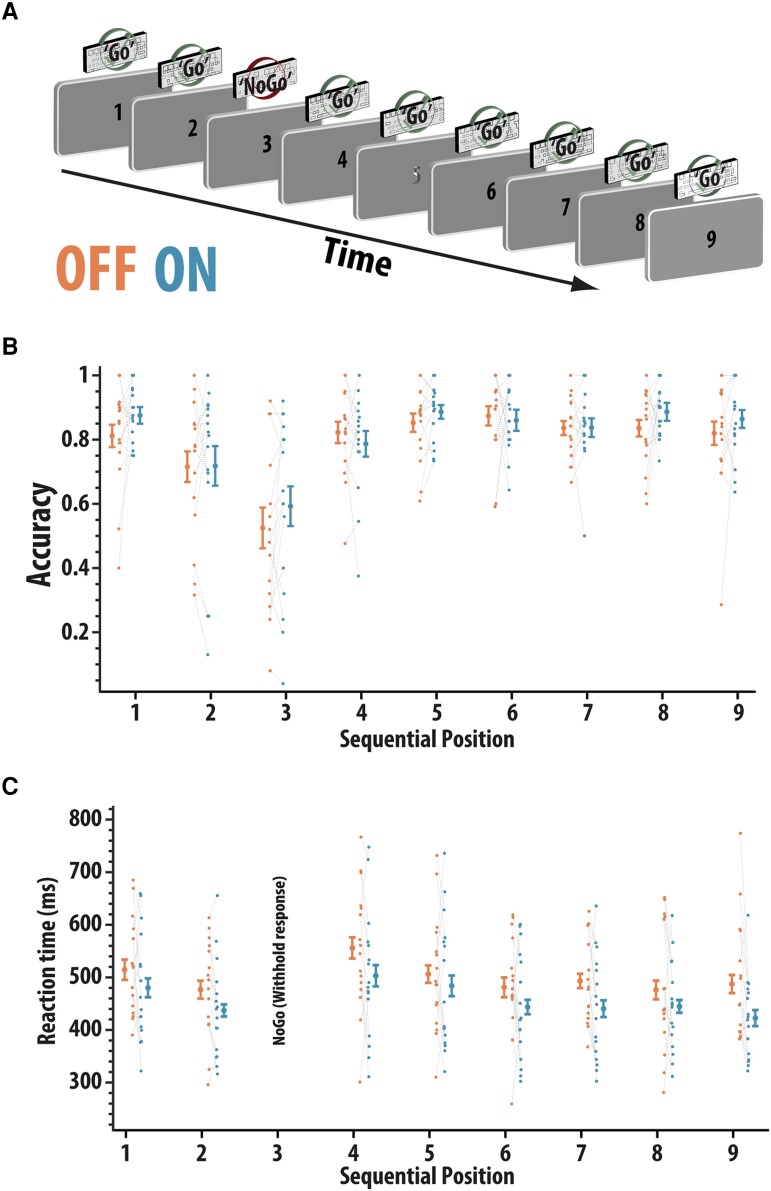
Sustained attention response task (SART). (**A**) Participants (*n* = 16) were presented with a fixed, repeating set of ascending digits (1–9) and had to make a button press to every digit apart from 3 when they had to abstain from making a response. (**B**) Accuracy (proportion correct) according to position in the sequence and drug status (ON and OFF rivastigmine). No significant effect of medication [ANOVA, *F*(1,15) = 1.36, *P* = 0.26]. Error bars reflect within-condition standard error. (**C**) Reaction times (ms) for correct responses split according to digit order and drug status (recall that participants should not press when presented with number 3, so there are no data here). Response times significantly reduced ON compared to OFF medication [ANOVA, main effect of drug; *F*(1,15) = 5.36, *P* = 0.035]. Error bars reflect within-subject standard error

Sustained attention was measured using a fixed-order variant of the sustained attention to response task (SART).^41,42^ Participants were presented with a fixed, repeating order of numbers (ascending 1–9). They had to make a button press every time a number appeared on the screen, with the exception of the number 3, in which a response had to be omitted (Fig. 1A). There were 225 trials in total. Response latencies and accuracy of responses (‘hits’ or ‘misses’) were recorded as the main dependent variables.

##### Precision spatial span task

**Figure 2:**
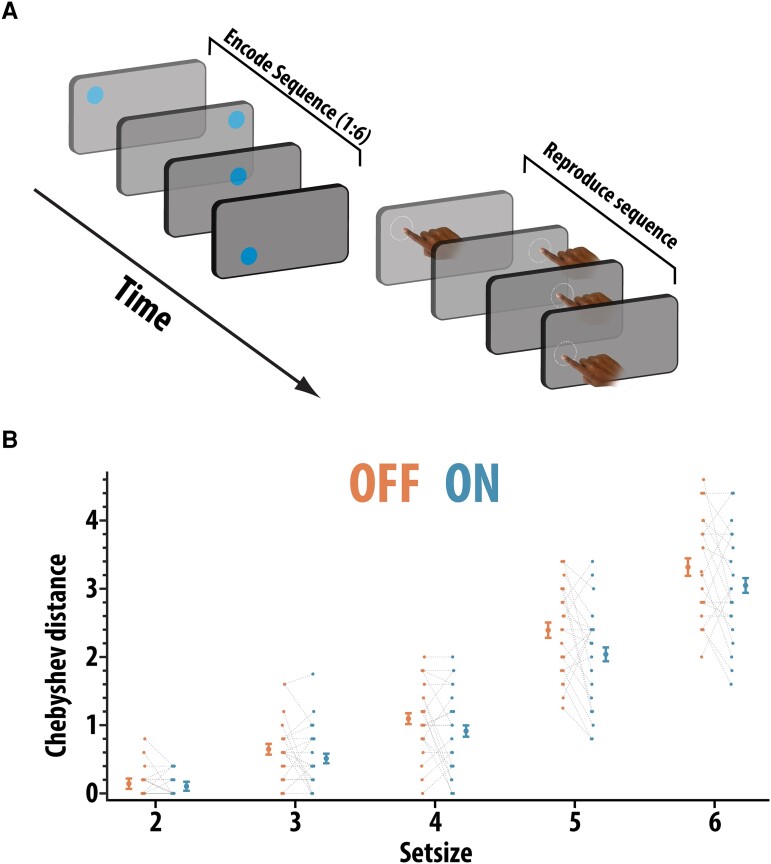
Precision spatial span task. (**A**) Participants (*n* = 21) had to remember the spatial locations of a series of dots presented sequentially on the screen (set size varied between 1 and 6) and then reproduce this sequence by touching the screen in the locations where the dots appeared. (**B**) Average Chebyshev distance (maximal displacement between response and target position. For example, if the third response was closest to the sixth presented item, this would be a Chebyshev distance of 3). Chebyshev distance was significantly reduced ON compared to OFF medication (ANOVA, main effect of drug; *F*(1,20) = 6.19, *P* = 0.022). Error bars reflect within-condition variance

Working memory spatial span has been found to be impaired in PD, particularly in patients with co-occurring executive deficits.^43,44^ The paradigms used to investigate spatial memory in PD patients have usually required participants to remember discrete spatial locations and then tap out their recollection of these to provide a binary measure of accuracy. Here, we probed the precision of their spatial working memory. Participants had to retain the locations of series of dots that appeared consecutively on the screen at different spatial locations (Fig. 2A). Then, they had to reproduce this spatial sequence by touching the screen at the locations in which they recalled the dots to have appeared, in the order in which they had appeared. Note, participants had to make the same number of responses as there were items before the trial ended, i.e. for a trial with a set size of four, the participant needed to make four responses to complete the trial. Our first main dependent variables on this task were average error distance for each set size. Average error was calculated as the pairwise Euclidean distance between the relevant actual target locations and the response locations according to the following equation:


AverageError=∑i=1N((responseXi−locationXi)2+(responseYi−locationYi)2)N

where *X* is the position on horizontal axis and *Y* is the position on the vertical axis and *n* is the set size. The number of spatial locations (set size) that had to be remembered varied from 1–6 (set sizes were intermixed). There were 30 trials in total.

Although the average error provides an indication of the overall accuracy of memory on each trial, it does not give much information about the extent to which memory has become corrupted. Examining the pattern of errors people make during recall can uncover the mechanisms responsible for these differences. Accordingly, we calculated the extent to which there was evidence of remembering the spatial locations, but reporting them in the incorrect order. Algorithmically, this was done by pairing each response location to its closest target location (i.e. nearest neighbour), such that every response location is assigned to one of the presented locations. We can then assign a number to each of the response locations corresponding to the order it appeared in the presented locations. For example, if in the six-location condition, the third and sixth locations were swapped, the response sequence would be 1 2 6 4 5 3. The Chebyshev distance (maximal displacement) can be used to provide a simple metric to quantify the extent to which the sequence was displaced, i.e. (6–3) = 3.

##### Simple one-item delayed short-term memory reproduction task

**Figure 3:**
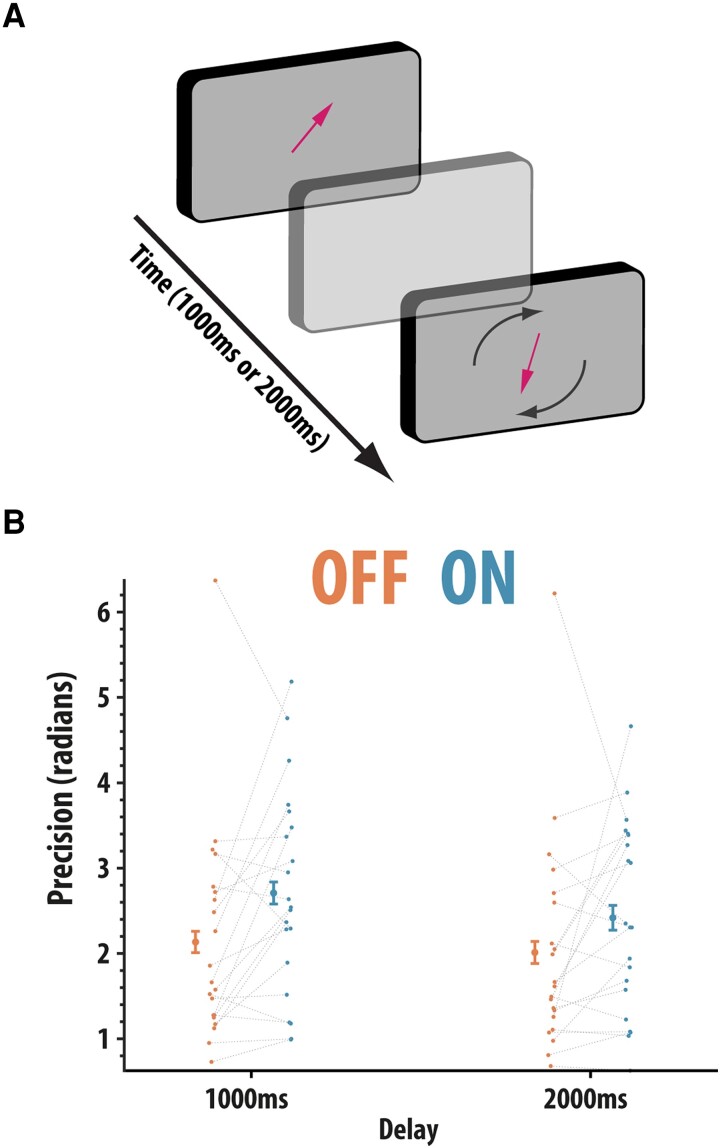
One-item delayed reproduction task. (**A**) Participants (*n* = 21) were presented with a single arrow presented at the centre of the screen. Participants were instructed to remember the orientation of this arrow because, after a variable delay (1000 ms or 2000 ms), participants had to reproduce the orientation of the previously presented arrow. (**B**) Precision (1/circular standard deviation) split according to delay period and drug status. Precision was significantly higher ON compared to OFF rivastigmine (ANOVA, main effect of drug; *F*(1,20) = 9.74, *P* = 0.005). Error bars reflect within condition standard error of the mean

The basic mnemonic abilities of patients was assessed using a simple delayed reproduction short-term memory task that has been used previously.^46^ In short, the orientation of a single, centrally presented arrow has to be remembered and reproduced after a brief variable delay period (1000 ms or 2000 ms; Fig. 3A). Recall was assessed by asking participants to reproduce the orientation of the previously presented arrow (e.g. by rotating the probe arrow clockwise (‘A’ key) or anti-clockwise (‘Z’ key) and pressing the ‘Space’ bar when finished). There were 48 trials for each delay duration (96 in total). The main dependent variable on this task was precision (see below).

##### One-item ignore/update task

**Figure 4:**
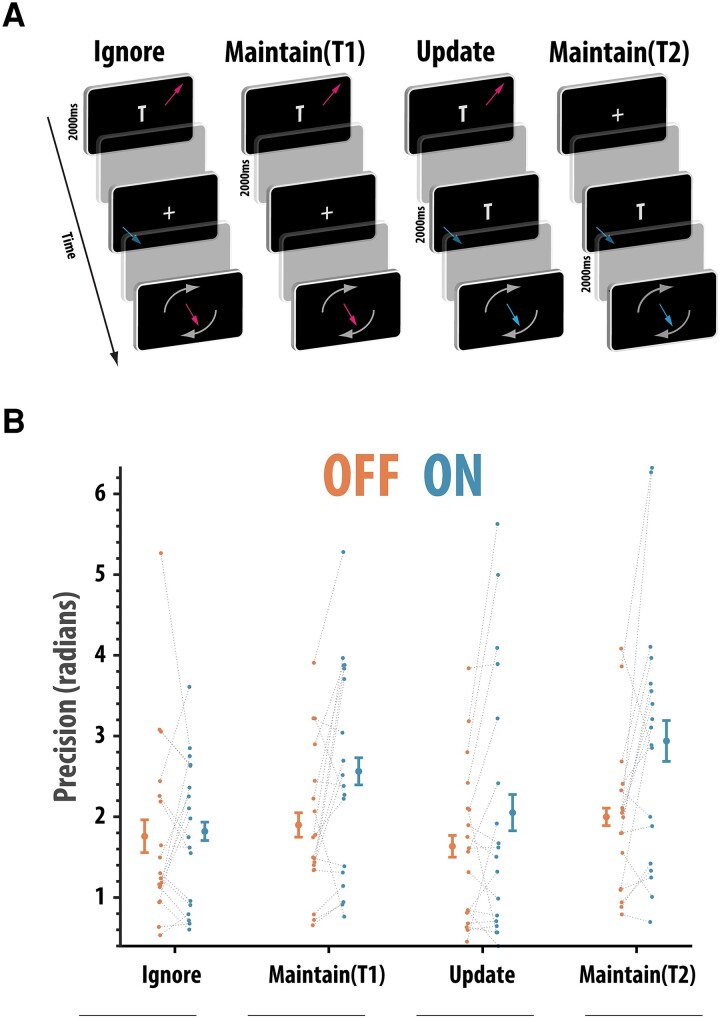
One-item ignore/update task. (**A**) The task requires participants (*n* = 18) to remember the orientation of one arrow across different circumstances and reproduce the orientation of this arrow. The ignore task required participants to ‘Ignore’ the orientation of an arrow presented during the delay period whereas the ‘Update’ task required this intervening arrow to be stored in memory and displace the previously presented arrow. There were two temporal controls [‘Maintain (T1)’ and ‘Maintain (T2)’] for the ignore and update conditions where the retention times were matched but in which no distracting information was presented. (**B**) Precision (1/circular standard deviation) split according to task type and drug status. Precision was significantly higher on the two maintain trials but not on the ignore and update trials (ANOVA, drug × presence of irrelevant information interaction; *F*(1,17) = 7.60, *P* = 0.040). Error bars reflect within condition standard error of the mean

The proficiency of ignoring and updating was a simplified (reducing the number of memoranda on any given screen by half) but otherwise identical task to that used to assess ignoring and updating.^47^ The method in which recall was probed was similar to the simple one-item delayed reproduction task (above), i.e. participants reproduced the orientation of an initially presented arrow after a delay period (Fig. 4A). Here, however, there were four conditions that varied in terms of which of the presented arrows had to be recalled and how long the delay period was between encoding and recall.

The ability to maintain items in memory but resist distraction was assessed by presenting a task-irrelevant item (another arrow) during the interval between encoding and probe (Ignore condition; Fig. 4A). A control trial type ‘without’ a distractor [‘Maintain (T1) condition’] was also included to act as a control so that the trial durations were matched.^47^ The inverse of the ignoring condition was also assessed. In this ‘Update condition’, rather than ignoring the information presented during the delay period between encoding and recall, the new arrow had to be stored in memory and the original encoded arrow discarded because it was now irrelevant. As in the Ignore condition, a temporally matched (2000 ms), control condition was also included [‘Maintain (T2)’].

The four conditions appeared in a randomized order. Rather than being explicitly cued about what item to retain, participants were instructed to only remember the last arrow presented with the letter ‘T’. (Figure 4A). Feedback was presented at the end of each trial to display the correct orientation of the arrow that had to be reproduced. Again, as this task was measuring the ability to reproduce orientations, the main dependent variable on this task was also precision (see below).

##### Attentional shifts in working memory task

**Figure 5:**
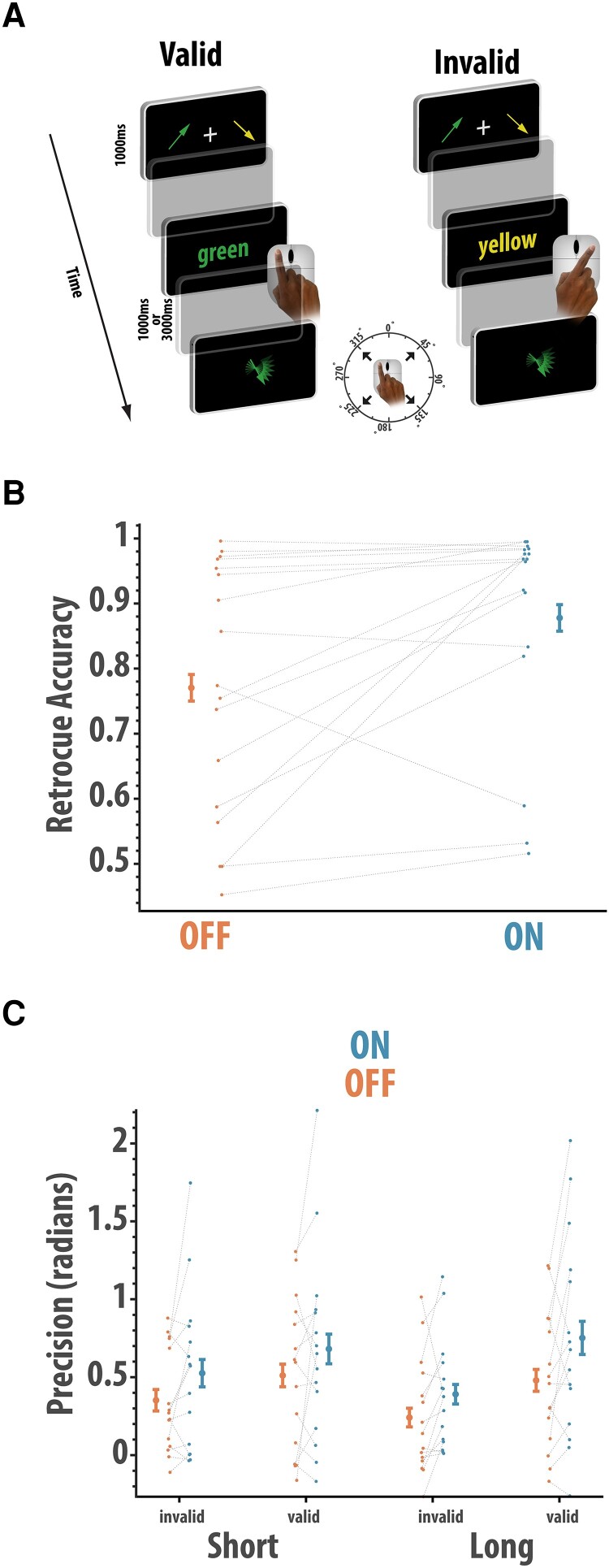
Effect of rivastigmine on shifting the focus of attention. (**A**) Participants (*n* = 16) remembered the orientations of two coloured arrows. During the delay, an incidental retro-cue probed their recall the about the location of one item (color cue). At the end of the delay, they reported the orientation of an arrow that could either be the same as the item interrogated previously (‘valid’) or the other item in memory (‘invalid’). (**B**) Accuracy of responding to the retro-cue as a function of rivastigmine. Patients ON medication had higher accuracy than patients OFF medication (Wilcoxon paired *t*-test; *W* = 21, *P* = 0.007). (**C**) Precision (1/SD) of response to the final arrow probe (*n* = 14), with higher scores reflecting more accurate recall, as a function of task condition and rivastigmine status. Precision was higher in patients ON rivastigmine compared to OFF rivastigmine (ANOVA, main effect of drug; *F*(1,14) = 5.01, *P* = 0.042). Error bars reflect within condition standard error of the mean

We measured how rivastigmine affects the ability to shift attention between items ‘held in memory’. In this task, two items had to be remembered over a delay, but a secondary ‘incidental’ task that required a shift of attention to one of the retained items, but was irrelevant, had to be performed (Fig. 5A). Two arrows of different colours and random orientations were shown, one on the left and one on the right of the screen, for 1000 ms. After a 500-ms blank screen, a ‘retro-cue’ was presented at screen centre, indicating a colour corresponding to one of the two arrows. This ‘incidental’ task thus required a shift of attention to one of the items held in memory. Participants clicked the left or right mouse button to indicate the side of the screen where they remembered the arrow corresponding to the cue colour had been shown. After a further delay of either 1000 ms or 3000 ms, the memory probe, a randomly oriented arrow, was presented. Another colour was shown at the centre of the screen, which could either be the same as the previously probed ‘incidental’ item (validly cued) or the other item (invalidly cued). Participants had to move their mouse in the direction in which they remembered the corresponding arrow was pointing. This enabled us to measure memory precision when the probed item was the same or different to the item that they had just paid attention to.

For memory tasks that required the reproduction of an orientation (simple one-item delayed reproduction task, one-item ignore/update task and attentional shifts in working memory task), our main dependent variable was precision (1/circular standard deviation adjusting for chance level performance). This was calculated according to the JV10_error function.^48^ In total, the tasks took ∼2 to 3 hours per participant, split up by two breaks. Participants also performed a saccadic task and a learning task not reported here.

##### Procedure

Patients attended on two sessions, once ON and once OFF rivastigmine, approximately two weeks apart. The order of these sessions was counterbalanced across individuals, in a randomized crossover design. On the OFF day, those taking twice-daily oral doses omitted their morning dose on the day of testing and the night-time dose the day before testing. Participants taking patches (*n* = 3) removed it at lunchtime on the day prior to their OFF testing session, due to time constant associated with transcutaneous absorption being longer.^49^ Three patients had their OFF session before commencing rivastigmine, and were therefore in a drug-naïve state for the OFF session. Overall, when ON, patients were tested on average 122 ± SD 44 minutes after their last dose (for patients on oral medication; for patients using patches, the patch remained on throughout). When OFF, they were tested on average 23.4 ± 3.1 hours after the last dose (for patients who had already been on the drug). The mean daily dose of rivastigmine was 6.3 ± SD 3.7 mg.

##### Statistical analysis

The data were analysed using R (3.6.3) in RStudio (1.2.5003). We used the Aligned rank transform package^50^ to perform non-parametric ANOVAs.

## Results

### Rivastigmine speeds responses on SART

Data on the SART task were available in 16 patients. We examined the effect of rivastigmine withdrawal on SART in which patients had to press a button in response to each number in a predictable, fixed-order digit stream (1–9), except when the number 3 was presented (Fig. 1A). The accuracy was examined in a 2 × 9 non-parametric repeated measures ANOVA with drug state (ON and OFF) and sequential position (1–9) as within-subject factors (Fig. 1B). Significant differences in accuracy were found according to sequential position [*F*(4.37,65.3) = 6.27, *P* < 0.0001]. There was no significant main effect of medication [*F*(1,15) = 1.36, *P* = 0.26] or interaction between drug and sequential position [*F*(4.5,68) = 1.04, *P* = 0.399]. Thus, there was no evidence that rivastigmine withdrawal affected the accuracy on this sustained attention task.

Next, we examined the effects of rivastigmine withdrawal on response latency for each of the eight stimuli for which a response was required, in a 2 × 8 non-parametric repeated measures ANOVA with drug (ON and OFF) and sequential position (1 to 8) as within-subject factors. Reaction times were found to vary significantly according to sequential position [*F*(3.4,52) = 4.43, *P* = 0.005; (Fig. 1C)]. Patients responded significantly quicker when ON rivastigmine compared to OFF [*F*(1,15) = 5.36, *P* = 0.035]. There was no evidence that the effect of drug varied by sequence (*F* < 1). Thus, cumulatively, rivastigmine showed evidence of enhancing the vigour of responding during a sustained attention task but was not found to affect the overall accuracy.

### Rivastigmine improved the integrity of spatial short-term memory

Next, we examined the effect of rivastigmine withdrawal on spatial short-term memory. Data were available in 21 subjects. In the spatial span task, participants had to remember the locations of series of dots presented sequentially on the screen (set size varied between 1 and 6) and then reproduce this sequence by touching the screen in the locations where the dots appeared.

First, we examined the overall error (average Euclidean distance between the target and response sequences) using a 2 × 6 non-parametric repeated measures ANOVA with drug state (OFF and ON) and set size (1 to 6) as within-subject factors. There was a trend towards a significant reduction in overall error between the ON compared to the OFF rivastigmine state [*F*(1,20) = 3.75, *P* = 0.067], whereby patients ON rivastigmine tended to have a shorter average distance between their responses and the spatial location of the memoranda. As expected, the error increased linearly with set size [*F*(3.76,75) = 132, *P* < 0.001], but did not significantly interact with medication (*F* < 1).

We next examined the extent to which participants’ responses occurred at the correct spatial locations (as defined by nearest neighbour, see ‘Methods’) but were produced in the wrong order (swap errors) using the Chebyshev distance as summary metric (Fig. 2B). A 2 × 5 repeated measures non-parametric ANOVA with drug state (OFF and ON) and set size (2 to 6) as within-subject factors revealed that Chebyshev distance (indexing the level of sequence displacement) was significantly higher in patients OFF rivastigmine compared to ON rivastigmine [*F*(1,20) = 6.19, *P* = 0.022]. Thus, when OFF rivastigmine, patients were more likely to reproduce the spatial locations in the wrong order to which they were presented. As expected, Chebyshev displacement distance increased with set size [*F*(3.1,63.6) = 374.6, *P* < 0.001], but there was no evidence for an interaction between rivastigmine and set size [*F*(2.46,49) = 1.2, *P* = 0.31]. Thus, cumulatively, cholinergic state predominantly affected sequence displacement (Chebyshev distance). Again, poorer performance (increased sequence displacement) was observed OFF rivastigmine compared to ON rivastigmine.

### Rivastigmine significantly boosts recall performance even for brief delays

We next examined the precision of short-term memory using a simple one-item delayed response task (Fig. 3A). Data were available for 21 patients. Precision data (1/circular standard deviation) were analysed using a non-parametric factorial ANOVA (see ‘Methods’) with drug (OFF and ON) and delay (1000 ms and 2000 ms) as within-subject factors. Precision was significantly improved ON compared to OFF drug [*F*(1,20) = 9.74, *P* = 0.005], i.e. there was less variation in the errors patients made ON, compared to OFF, rivastigmine. Precision also significantly worsened with delay period from 1000 ms to 2000 ms [*F*(1,20) = 5.42, *P* = 0.03; Fig. 3B]. However, the effects of drug did not significantly vary according to delay [*F*(1,20) = 1.27, *P* = 0.27]. A confirmatory analysis in which we examined precision by collapsing across delay also revealed that drug significantly improved precision (Wilcoxon paired test, *W* = 46, *P* = 0.014, rb = 0.60). In summary, data from this task show that precision was strongly affected by cholinergic state: worse performance (precision) was seen in the OFF compared to the ON state.

### Distractors annul the beneficial effect of rivastigmine

Next, the ability either to successfully ignore irrelevant information during the delay period, update information currently held in memory or simply maintain it across longer delays (2000 ms versus 6000 ms) was evaluated (Fig. 4A). Data were available for 18 patients. First, we examined precision (1/SD adjusting for chance using JV10 fit). A 2 × 2 × 2 non-parametric (rank-based) ANOVA with drug (OFF and ON), duration (long, short) and presence of irrelevant information (present and absent) as within-subject factors was performed. Precision was significantly worse on trials that contained irrelevant information compared to maintain only trials [*F*(1,17) = 18.51, *P* < 0.001], but there was no significant effect of delay on precision (*F* < 1). Overall, patients ON rivastigmine had better precision than when OFF rivastigmine [*F*(1,17) = 5.96, *P* = 0.026]. However, significant variation in the positive effects of drug was found according to the presence or absence of irrelevant information [*F*(1,17) = 7.50, *P* = 0.014].

Non-parametric paired comparisons revealed that when ON rivastigmine, patients had improved precision on trials that ‘only required maintenance’ (*W* = 19, *P* = 0.002, rb = 0.77), but there was no such significant effect for trials that contained irrelevant information, i.e. when either ignoring or updating was required (*W* = 74, *P* = 0.64, rb = 0.13). There was no significant interaction between delay and the presence of irrelevant information [*F*(1,17) = 2.01, *P* = 0.17]. No other effects were significant (*F*s < 1). Thus, cumulatively, the findings from this task indicate that rivastigmine withdrawal selectively affected performance on trials involved in maintaining information, but crucially not on trials that also required irrelevant information to be ignored or updated.

### Rivastigmine withdrawal impairs working memory independently of any need to shift the focus of attention

Next, we examined whether rivastigmine affects the benefit from focusing attention on one item in memory. In this paradigm (Fig. 5A), a retro-cue required participants to identify the location of one of the items during the retention delay (e.g. a green cue would require recall of whether the green item was on the left or right in the previously presented display). Data were available for 16 patients. Firstly, we examined participants’ performance on the incidental retro-cue task using a Wilcoxon paired test. Patients ON rivastigmine (*M* = 0.87, SD = 0.19) were significantly more accurate compared to patients OFF rivastigmine (*M* = 0.77, SD = 0.16) in correctly identifying the location on the screen in which the cued colour appeared (*W* = 21, *P* = 0.007, rb = 0.72). Thus, the basic short-term maintenance of colour-location binding was significantly impaired in patients OFF rivastigmine (Fig. 5B).

We next examined the precision (1/SD) according to drug (Fig. 5C), validity and delay using a non-parametric repeated measures ANOVA (only for those trials where the response to the retro-cue was correct, although similar effects of rivastigmine withdrawal were observed if all data, from correct and incorrect retro-cue trials, were used). Two participants did not have sufficient trials in all conditions and were excluded from the analysis. This analysis revealed that there was a significant main effect of rivastigmine [*F*(1,14) = 5.01, *P* = 0.042] with patients ON showing higher precision than when OFF. There was a trend towards valid trials having higher precision compared to invalid trials [*F*(1,14) = 3.81, *P* = 0.07]. There was no significant difference in recall according to delay [*F*(1,14) = 2.07, *P* = 0.17], and there was a trend towards an interaction between delay and validity [*F*(1,14) = 3.61, *P* = 0.07]. None of the other effects were significant (*F* < 1). Thus, as in the other indices of short-term recall, patients ON rivastigmine showed superior performance (accuracy and precision) compared to those OFF rivastigmine. There was no evidence that this varied with the need to shift the focus of attention or delay.

## Discussion

The current study examined how pervasively rivastigmine affects cognitive functioning in people with dementias associated with Lewy body pathology—PDD and DLB—using a novel withdrawal design. It soughts to address whether rivastigmine has a selective effect on attention or also produces gains on a variety of putatively distinct short-term or working memory components. A global decline in attention was found, as indexed by increased reaction time on the SART sustained attention task (Fig. 1), after rivastigmine withdrawal. However, rivastigmine had no effects on cognitive control on this paradigm as assessed by the ability to withhold a response (inhibitory control). Across all tasks measuring short-term memory (Figs 2–5), impairments in recall were observed when rivastigmine was withdrawn. However, these effects were load- and delay-independent suggesting that acetylcholinesterase inhibition boosts the processing of visual stimuli generally (attentional effect) rather than boosting mnemonic performance (Figs 2–4). There was also no evidence that rivastigmine significantly affected performance according to the requirement to shift the focus of attention in memory when prompted by retro-cues (Fig. 5). Contrary to the generally ameliorative effects of rivastigmine on short-term recall, improvements were not observed on demanding tasks requiring top-down control over the contents of working memory (ignoring and updating; Fig. 4). Cumulatively, these data support the notion that cholinergic augmentation predominantly enhances visual attention and that these gains may feed through to improvements on tests of short-term memory (though, we recognize that the data could support other interpretations, discussed below). In contrast, performance improvements were neither evident when complex manipulation of information was required (ignoring and updating), nor were there specific interactions between cholinergic state and the effect of delay, load or shifting the focus attention prompted by retro-cues, or when responses had to be withheld on the SART.

These findings contrast with the results of studies that have examined the effects of dopamine withdrawal on cognition. For nearly 40 years, dopaminergic medication withdrawal designs have been used to understand the dopamine-dependent nature of psychological deficits in PD.^35,36,51–57^ There are multiple reports of attention (set shifting) being unaffected by dopamine withdrawal in non-demented PD patients^34,52,58^ (see Fallon^59^ for a discussion) and only diminishing the willingness to engage in an attentionally demanding Rapid Serial Visual Processing task.^60^ Thus, the present finding of worse sustained attention after rivastigmine withdrawal points to cholinergic systems supporting attention in ways that dopamine does not. Similarly, the uniformly positive effects of the high cholinergic state on short-term recall are a stark departure from what has been observed in dopamine withdrawal studies in which there is evidence for a nuanced, task-dependent role of dopamine in short-term recall. First, the effects of dopamine vary by domain, e.g. deficits on spatial but not verbal working memory in the OFF dopamine state.^36,61^ Second, deficits OFF dopamine more readily appear when information needs to be controlled in working memory.^34,47,62^ A starker contrast between the effects of rivastigmine and dopamine withdrawal on working memory can be gained from considering the multitude of observations that dopamine withdrawal can ‘improve’ working memory, but only in certain contexts.^38,63–65^ Again, this would suggest that cholinergic augmentation has the capacity to improve cognition in ways beyond that which can be achieved by dopaminergic enhancement.

### Role of cholinergic augmentation in modifying attentional deficits

Fluctuating attention is a significant problem in PDD and DLB, associated with increased incidence of negative clinical features such as falls and reduced quality of life.^66,67^ Despite prior evidence for a positive effect of rivastigmine on sustained attention,^6,11^ the neurocognitive mechanisms underlying attentional impairments have not been fully established. Here, using a test of sustained attention (SART;^41,42^) general impairments after rivastigmine withdrawal were found (reduced response latency)]. A key component of the SART is the need to withhold a response. Failure to do this (commission errors) has been reported to be affected by catecholamine-boosting drugs like methylphenidate in patients with attention deficit hyperactivity disorder (ADHD).^68^ In this study, reductions in commission errors did not differ significantly according to cholinergic status (ON or OFF rivastigmine). Thus, there was no evidence that rivastigmine has any specific effects on cognitive control on this task (e.g. reduced commission errors due to altered inhibitory control) on attention.

It could be argued that because this study found only that rivastigmine significantly affected response latency and not accuracy that the results do not reflect a genuine enhancement of sustained attention. Previous reports have suggested that individuals performing the SART can make strategic shifts along a speed–accuracy axis, which are independent and distinct from sustained attention.^69^ However, in our study, there was no evidence for a speed–accuracy trade-off. There was no significant effect of drug status on accuracy. Indeed, numerically, patients ON rivastigmine tended to perform at a higher level than patients OFF rivastigmine. Thus, the results suggest that rivastigmine uncouples the normally inverse relationship between speed and accuracy. Computational models of how this process occurs have proposed several factors that may drive this effect, such as decreased neuronal noise, a process dependent upon neurotransmitters such as dopamine.^70^ However, future investigations will be needed to isolate the specific mechanisms through which rivastigmine can enhance attention.

The present study used a fixed version of the SART, where the requirement to withhold a response occurs at a fixed point in an ascending sequence. The fixed version has greater sensitivity to detect impairments in clinical groups^41,42^ and provokes greater cortical engagement.^42^ However, future studies might profitably examine the effect of rivastigmine where there is greater ‘intermixing’ between ‘Go’ and ‘NoGo’ trials. For example, converging cross-species work^71^ has indicated that cholinesterase inhibition affects performance during specific attentional circumstances. During a sustained attention task in which Go and NoGo trials were intermixed, cholinergic transients (or putative human analogues detected with neuroimaging) were found to be generated, not according to whether a Go or NoGo trial was being performed, but to the Go trials that followed from failed (response was made) NoGo trials (labelled ‘incongruent hits’).^71^ Thus, the absence of specific effects of rivastigmine on NoGo trials might potentially be due to the fixed nature of the SART task deployed here.

### Delay and load-independent effects on recall after rivastigmine withdrawal

In addition to sustained attention, this study also sought to determine whether rivastigmine withdrawal impaired other cognitive functions, such as short-term recall and various putatively distinct sub-components of cognitive control over the contents of memory (delay, load, ignoring, updating and shifting the focus of attention). Collapsing over all conditions, short-term recall was generally impaired after rivastigmine withdrawal, illustrating that cognitive gains after cholinesterase inhibition extend into the mnemonic domain. However, these positive effects may not be attributable to improved short-term memory *per se* and may arise merely as the consequence of improved attentional performance. For example, many theories have argued that mnemonic performance, across multiple timescales and through different mechanisms, is fundamentally yoked to attentional performance.^72–74^ This coupling may arise due to the mundane fact that information needs to be attended to in order to be remembered or due to more contested ideas, such that there is a homology between internal and external attention, i.e. the same cortical regions involved in perceiving information are also involved in maintaining representations of that information recall.^75–79^

Moreover, although improvements on short-term recall after rivastigmine were found, these effects were crucially delay and load-independent, i.e. the gains from cholinesterase inhibition did not scale with the delay period or set size. This provides important clues concerning the underlying neurocognitive mechanisms of this effect. Animal work suggests that reductions in cholinergic signalling across the cortex [nucleus basalis of Meyernet (nBM)] can produce delay-independent effects, whereas lesions to the septo-hippocampal cholinergic system produce delay-dependent deficits.^18,80^ Mapping this division of labour onto the current study, it is possible to speculate that rivastigmine improves the precision of recall through acting on cortical, rather than hippocampal, circuits.

Given the delay/load-independent nature of the improvements on our short-term memory tasks, it is tempting to conclude that rivastigmine produced these improvements vicariously, through enhancing attention (perhaps via modulation of neuronal circuits in receipt of cholinergic fibres originating in the nBM). However, there are other viable explanations.

The requirement to show that neural perturbations exert delay-dependent effects has frequently been deployed to establish the mnemonic, as opposed to perceptual or motoric, nature of these effects e.g.^81^ seminal findings in the short-term memory literature have demonstrated delay-dependent effects, for example, dopaminergic manipulations only affecting memory when tasks include a delay between encoding and response.^82^ Traditional models of working memory have placed particular emphasis on the persistent firing of prefrontal neurons as a defining feature of short-term memory.^83^ Within that framework, inducing delay-dependent effects, perhaps via altered delay-period firing,^84^ after rivastigmine could have been expected. The absence of delay-dependent effects and the presence of an effect of the drug on attention could point towards attentional modulation as the origin of rivastigmine’s effects.

However, prefrontal neurons can support working memory through distinct and dynamic mechanisms.^85^ Thus, there are a diverse set of ways that rivastigmine could affect recall-supporting prefrontal neurons that would not necessarily produce delay-dependent effects, e.g. reactivation of memories. Similarly, there are empirical demonstrations of cholinergic perturbations producing delay-independent effects that are not attentional in nature.^86^ Thus, there are precedents for the contention that there is a non-attentional route through which cholinergic changes can affect working memory in a delay-independent manner. Finally, it should also be acknowledged that there might be greater disparity in what researchers regard as an attentional effect as opposed to a mnemonic effect.^20,77,78,87^ Empirical, rather than semantic, concerns should take precedence in the form of neuroimaging studies that examine where, when and under what conditions rivastigmine affects cerebral activity. Indeed, cholinergic drugs have been found to exert different effects on encoding and retrieval of information.^12^

### Rivastigmine prevents order of mnemonic information from becoming corrupted

One aspect of the results that does suggest that rivastigmine can have specific modulatory effects on memory is its effects on misbinding during recall. In the present study, the overall effect of rivastigmine on the precision of spatial memory was weak, i.e. there was no significant effect of drug on the error distance of responses. Rather, the effects of rivastigmine were most pronounced when examining the type of memory errors participants made (Fig. 2). Specifically, cholinergic state significantly affected the tendency to incorrectly reproduce the order of the to-be-remembered spatial pattern (as indexed by Chebyshev distance). In other words, the memory for the presented spatial locations themselves was retained, but they were reproduced in the incorrect order. Therefore, there was evidence that the ability to correctly bind or retrieve information in the correct, uncorrupted order could be improved by rivastigmine.

Previous reports have found that non-demented PD patients can be distinguished from healthy age-matched controls by the tendency to make more misbinding errors at higher loads.^45^ Given prior work demonstrating that misbinding errors are increased in patients with lesions to the hippocampus,^88,89^ it might be speculated that the withdrawal-induced increase in misbinding occur due to modulation of the same neural locus. However, in a non-mutually exclusive fashion, the result could also occur vicariously through the effect rivastigmine has on attention. Attention has long been theorized to be necessary to enable binding to take place^90^ and play a key role in visual working memory.^91,92^ Thus, increased misbinding after rivastigmine withdrawal may also be a downstream consequence of impaired attention.

### No evidence that rivastigmine specifically improves control over working memory

Further clues about the neurocognitive effects of rivastigmine can be found by looking at the unequal effects withdrawal had on different mnemonic control sub-processes, which have been argued to have separate neural components.^73,78,87^ This study tested three different forms of manipulating information in short-term memory: ignoring, updating and shifting the focus of attention. With regard to ignoring and updating, we used a design^47^ that allowed us to examine whether rivastigmine exerted antagonistic or mutually beneficial effects on ignoring and updating whilst controlling for the confounding effects of delay.

Patients ON rivastigmine improved on maintain only trials, i.e. where no task-irrelevant information had to be ignored or jettisoned (updated). However, there was no corresponding improvement on ignore or update trials. This indicates that the beneficial effects of cholinesterase inhibition do not extend to all short-term memory tasks. Specifically, cholinergic augmentation does not seem to improve the capacity to protect short-term recall from interference. This accords well with the above discussion that rivastigmine improves short-term recall in a delay and load-independent manner as these are also processes putatively requiring the control of interference.^93^

A large corpus of work implicates frontostriatal regions^94–98^ particularly under the guidance of dopamine^34,98–102^ in enabling people to control the contents of memory. Indeed, previous work has suggested that dopamine withdrawal in non-demented PD patients can impair ignoring and updating, without affecting the ability to maintain items.^47^ Thus, dopamine and acetylcholine appear to support different functions in short-term recall. As such, this finding provides novel support and refinement of the dual syndrome view,^4^ whereby dopamine (or monoamine) disruption to frontostriatal circuits produces deficits in the ‘executive’ control of memory, but cholinergic disruption to posterior, sensory regions produce impaired visual memory.

These findings may also extend to other forms of attentional manipulation in memory, such as the efficacy of shifting the focus of attention. Recent studies have led to the recognition that not all items in working memory are stored in the same way, and that some might be held in a so-called privileged state, sometimes referred to as the focus of attention.^103^ It has also been argued that such shifts may be associated with increased hippocampal involvement in storing information outside the focus of attention.^104,105,77^ The presentation of retrospective cues (retro-cues) after encoding has been found to enhance recall for the cued items at the expense of the non-cued items^103,106,107^ by pre-selecting information in memory, bringing it into a more active form, ready for action.^93^ Specifically, prior studies have found that requiring participants to make a perceptual judgement on previously seen memoranda can putatively bring the cued items into the focus of attention and improve recall.^108^ Here, we tested the effect of rivastigmine on the efficacy of this process. There was no evidence that cholinesterase inhibition specifically modulated this function, i.e. absence of a significant drug by validity interaction. Thus, shifting items into and out of the focus of attention does not appear to be under cholinergic support in DLB.

### Clinical implications and future work

The present results provide important information on the extent to which acetylcholinesterase inhibition will improve cognitive function. Whilst general improvements were found in visual attention across multiple paradigms, the results also suggest that there are several aspects of cognitive control or executive functioning that are not affected by acetylcholinesterase inhibition. This highlights the need for further interventions to improve cognitive symptoms in Lewy body dementias. However, although certain cognitive deficits were refractory to rivastigmine withdrawal, it is notable that improvements in certain cognitive functions were not observed, as is frequently reported after dopamine withdrawal.^52,62,109^

Another unresolved issue from this study is the interaction between dopamine and acetylcholine in contributing to the cognitive profile observed here. Here, no changes were made to whatever dopaminergic medication patients were taking, with the aim of isolating the specific contributions cholinergic stimulation made to cognition. However, there are substantial interactions between dopamine and acetylcholine and differing ratios of acetylcholine to dopamine may influence cognitive performance.^2^ Future experiments might profitably seek to fractionate the effects of dopaminergic drugs and rivastigmine by examining the effects of dopaminergic and cholinergic drug withdrawal at separate times, within the same patient. It has also been demonstrated that the effects of cholinergic drugs vary substantially across individuals and that degeneration of nucleus basalis of Meynert may predict the positive or negative effects of these drugs.^110^ Future studies might combine rivastigmine withdrawal with neuroimaging of midbrain regions to investigate this claim.

### Summary

Attentional improvements are well recognized with rivastigmine in PDD and DLB. However, whether all short-term memory, and its various sub-components, that is also affected remained unknown. Here, we provided evidence that rivastigmine is likely to improve attention and that this improvement co-occurred with general enhancement of short-term memory. However, control over items in memory appeared to be unaffected by drug withdrawal.

## Data Availability

Our ethics approval does not permit public archiving of the data supporting this study. Those wishing to access to this data should contact the lead author, S.J.F. Access can be obtained and granted to named individuals. To obtain the data, investigators must complete a formal data sharing agreement, including conditions for secure storage of sensitive data.
